# Supplementary material for: An intrinsic mechanism of metabolic tuning promotes cardiac resilience to stress
Source: EMBO Mol Med. 2024 Sep 13;16(10):2450–84. doi: 10.1038/s44321-024-00132-z (PMC11473679; doi:10.1038/s44321-024-00132-z)
Supplement: Supplementary file 1 — Table EV1 [file 44321_2024_132_MOESM1_ESM.docx]

**Table EV1**

| **Lipids ug/mg** | | | **wt129SV** | | **DS** | **Mel null** | **DS** | **wtFVB** | **DS** | **Mel over** | **DS** |
| --- | --- | --- | --- | --- | --- | --- | --- | --- | --- | --- | --- |
| Satured FAs | | | 4,569 | | 0,904 | 4,837 | 0,175 | 4,972 | 0,948 | 4,808 | 0,399 |
| mono-unsatured FAs | | | 1,280 | | 0,284 | 1,342 | 0,125 | 1,105 | 0,219 | 1,247 | 0,045 |
| poli-unsatured FAs | | | 7,574 | | 1,545 | 8,209 | 0,481 | 7,299 | 1,355 | 7,038 | 0,605 |
| ω-6 FAs | | | 3,655 | | 0,698 | 4,138 | 0,150 | 3,160 | 0,653 | 3,247 | 0,263 |
| ω -3 FAs | | | 3,919 | | 0,869 | 4,071 | 0,348 | 4,139 | 0,714 | 3,790 | 0,355 |
| total FAs | | | 13,423 | | 2,728 | 14,388 | 0,614 | 13,376 | 2,496 | 13,093 | 1,036 |
| C16 FAs | | | 2,122 | | 0,381 | 2,191 | 0,032 | 2,466 | 0,549 | 2,438 | 0,198 |
| C16:1 FAs | | | 0,098 | | 0,027 | 0,075 | 0,018 | 0,083 | 0,019 | 0,125 | 0,026 |
| C18 FAs | | | 2,447 | | 0,529 | 2,646 | 0,155 | 2,506 | 0,400 | 2,369 | 0,204 |
| C18:1 FAs | | | 1,182 | | 0,257 | 1,266 | 0,111 | 1,022 | 0,202 | 1,122 | 0,070 |
| C18:2 FAs | | | 2,674 | | 0,526 | 2,990 | 0,142 | 2,299 | 0,517 | 2,416 | 0,200 |
| C18:3 γ FAs | | | 0,029 | | 0,016 | 0,027 | 0,014 | 0,030 | 0,017 | 0,033 | 0,017 |
| C18:3 α FAs | | | 0,045 | | 0,010 | 0,050 | 0,006 | 0,041 | 0,010 | 0,049 | 0,007 |
| C20:3 FAs | | | 0,057 | | 0,014 | 0,066 | 0,007 | 0,073 | 0,020 | 0,062 | 0,001 |
| C20:4 FAs | | | 0,895 | | 0,155 | 1,054 | 0,033 | 0,758 | 0,136 | 0,737 | 0,052 |
| C20:5 FAs | | | 0,030 | | 0,008 | 0,025 | 0,002 | 0,032 | 0,007 | 0,029 | 0,002 |
| C22:5 FAs | | | 0,179 | | 0,042 | 0,171 | 0,011 | 0,193 | 0,050 | 0,223 | 0,022 |
| C22:6 FAs | | | 3,666 | | 0,818 | 3,825 | 0,332 | 3,873 | 0,660 | 3,489 | 0,328 |
| triglycerides | | | 0,712 | | 0,252 | 0,815 | 0,281 | 0,802 | 0,307 | 1,103 | 0,333 |
| C16 triglycerides | | | 0,419 | | 0,087 | 0,454 | 0,179 | 0,392 | 0,187 | 0,529 | 0,177 |
| C16:1 triglycerides | | | 0,083 | | 0,025 | 0,043 | 0,039 | 0,068 | 0,031 | 0,126 | 0,048 |
| C18 triglycerides | | | 0,056 | | 0,019 | 0,062 | 0,020 | 0,057 | 0,031 | 0,058 | 0,015 |
| C18:1 triglycerides | | | 0,337 | | 0,087 | 0,401 | 0,155 | 0,294 | 0,138 | 0,396 | 0,149 |
| C18:2 triglycerides | | | 0,309 | | 0,080 | 0,454 | 0,165 | 0,371 | 0,244 | 0,434 | 0,267 |
| C18:3γ triglycerides | | | 0,031 | | 0,007 | 0,016 | 0,004 | 0,036 | 0,007 | 0,034 | 0,004 |
| C18:3α triglycerides | | | 0,019 | | 0,008 | 0,020 | 0,006 | 0,016 | 0,008 | 0,023 | 0,012 |
| C20:3 triglycerides | | | 0,003 | | 0,001 | 0,005 | 0,004 | 0,004 | 0,002 | 0,004 | 0,001 |
| C20:4 triglycerides | | | 0,020 | | 0,011 | 0,015 | 0,009 | 0,012 | 0,007 | 0,017 | 0,016 |
| C20:5 triglycerides | | | 0,006 | | 0,007 | 0,004 | 0,002 | 0,004 | 0,005 | 0,003 | 0,002 |
| C22:5 triglycerides | | | 0,021 | | 0,014 | 0,015 | 0,012 | 0,015 | 0,011 | 0,021 | 0,017 |
| C22:6 triglycerides | | | 0,053 | | 0,031 | 0,051 | 0,036 | 0,062 | 0,041 | 0,072 | 0,065 |
| cholesterol | | | 0,484 | | 0,023 | 0,451 | 0,014 | 0,455 | 0,041 | 0,507 | 0,028 |
| esterified cholesterol | | | 0,016 | | 0,002 | 0,018 | 0,002 | 0,021 | 0,004 | 0,031 | 0,009 |
| cardiolipin | | | 3,143 | | 1,064 | 2,892 | 0,349 | 2,263 | 0,182 | 3,144 | 0,957 |
| C16 cardiolipin | | | 0,103 | | 0,011 | 0,100 | 0,021 | 0,102 | 0,018 | 0,092 | 0,022 |
| C16:1 cardiolipin | | | 0,046 | | 0,016 | 0,040 | 0,007 | 0,049 | 0,019 | 0,050 | 0,011 |
| C18 cardiolipin | | | 0,048 | | 0,003 | 0,041 | 0,005 | 0,038 | 0,014 | 0,042 | 0,015 |
| C18:1 cardiolipin | | | 0,174 | | 0,031 | 0,148 | 0,013 | 0,161 | 0,036 | 0,158 | 0,035 |
| C18:2 cardiolipin | | | 1,169 | | 0,283 | 1,162 | 0,096 | 1,061 | 0,262 | 1,057 | 0,091 |
| C22:6 cardiolipin | | | 0,071 | | 0,032 | 0,063 | 0,015 | 0,139 | 0,015 | 0,081 | 0,005 |
| LEGEND:  fatty acids (FAs), triglycerides, cardiolipin are indicated as C=carbon, number of carbons: number of double bonds, greek=isoform; ω -6= omega-6 fatty acids; ω -3= omega-3 fatty acids; |  |  | |  | | | | | | |  |
