# Supplementary figures and images for: An intrinsic mechanism of metabolic tuning promotes cardiac resilience to stress
Source: EMBO Mol Med. 2024 Sep 13;16(10):2450–84. doi: 10.1038/s44321-024-00132-z (PMC11473679; doi:10.1038/s44321-024-00132-z)

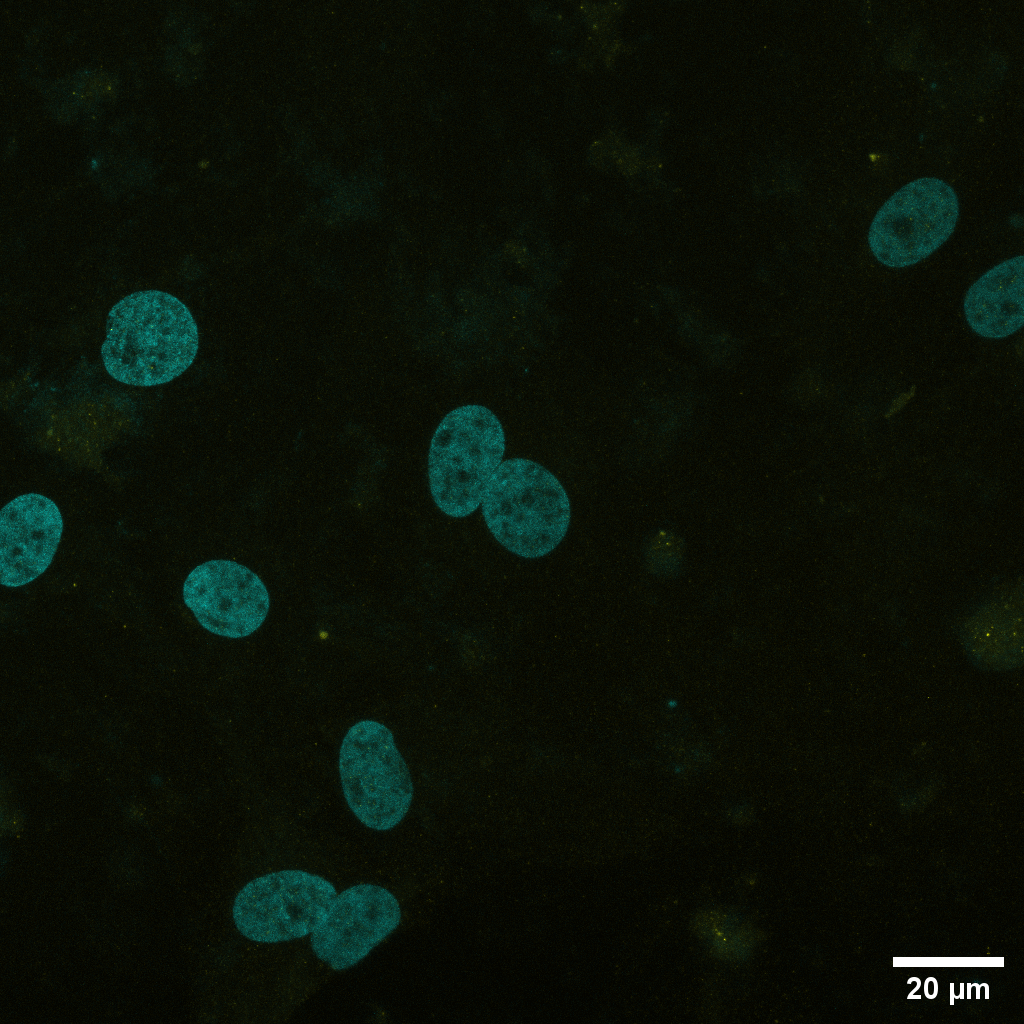

Supplement: Supplementary file 3 — Source data Fig. 1 [file 44321_2024_132_MOESM3_ESM.zip › Figure 1/1G/Max intensity_Mel null hiPSC-CMs_AAV6-empty_Myc IF.tif]

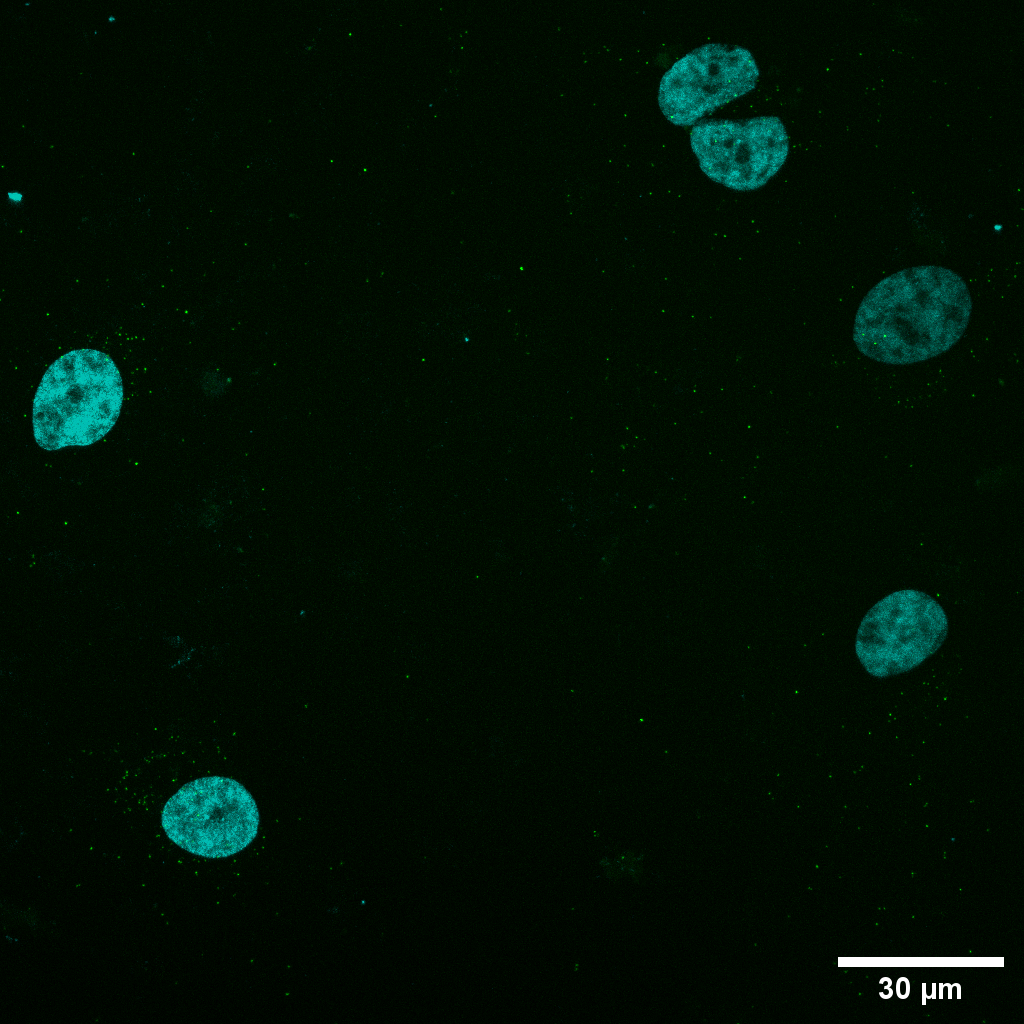

Supplement: Supplementary file 3 — Source data Fig. 1 [file 44321_2024_132_MOESM3_ESM.zip › Figure 1/1G/Max intensity_Mel null hiPSC-CMs_AAV6-empty_Myc+α-MTP PLA.tif]

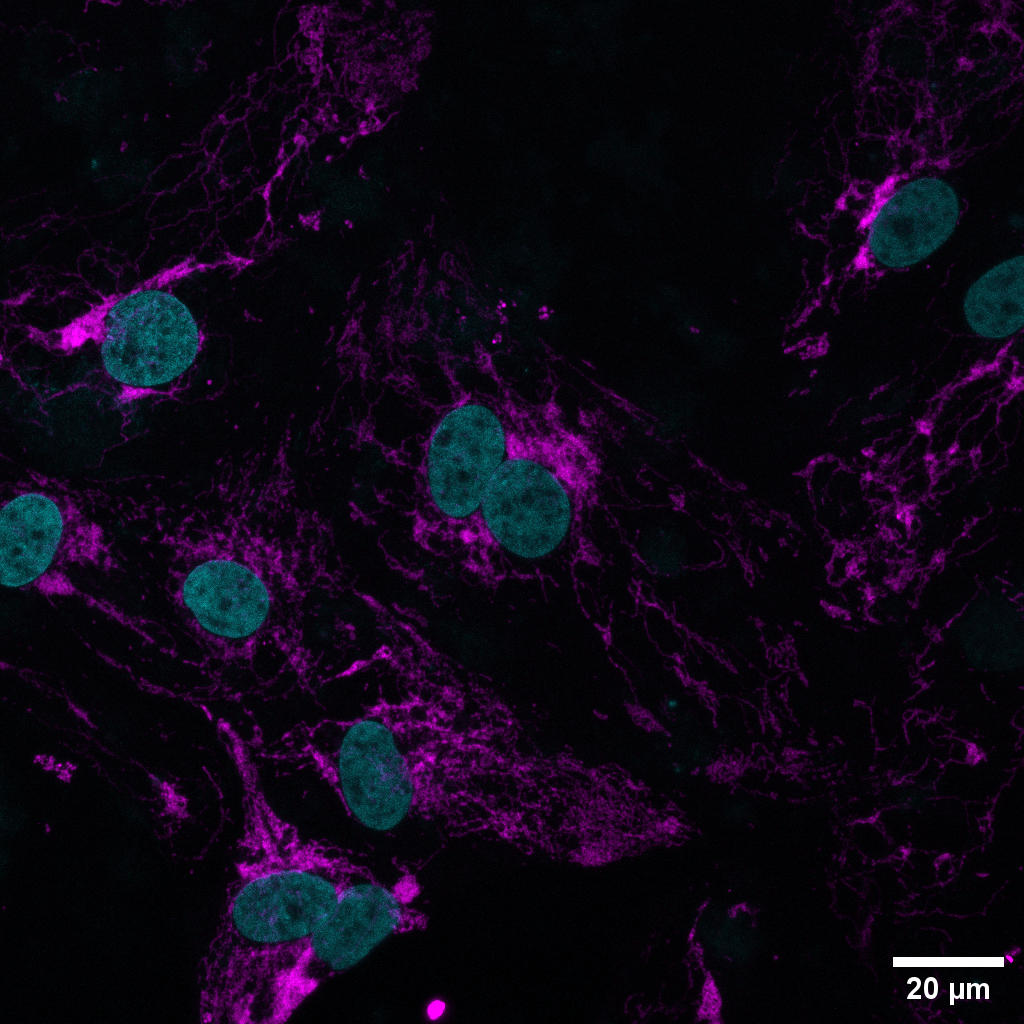

Supplement: Supplementary file 3 — Source data Fig. 1 [file 44321_2024_132_MOESM3_ESM.zip › Figure 1/1G/Max intensity_Mel null hiPSC-CMs_AAV6-empty_α-MTP IF.tif]

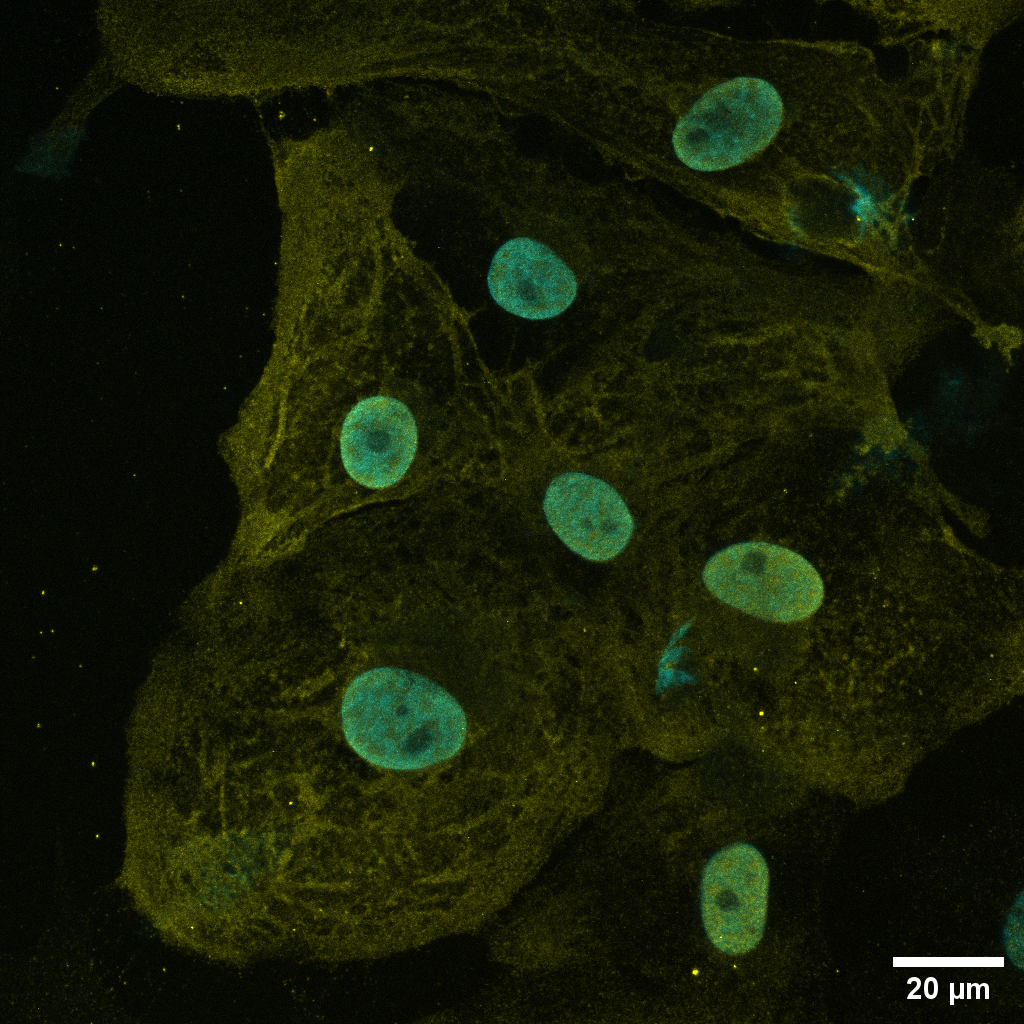

Supplement: Supplementary file 3 — Source data Fig. 1 [file 44321_2024_132_MOESM3_ESM.zip › Figure 1/1G/Max intensity_Mel null hiPSC-CMs_AAV6-Myc-Mel_Myc IF.tif]

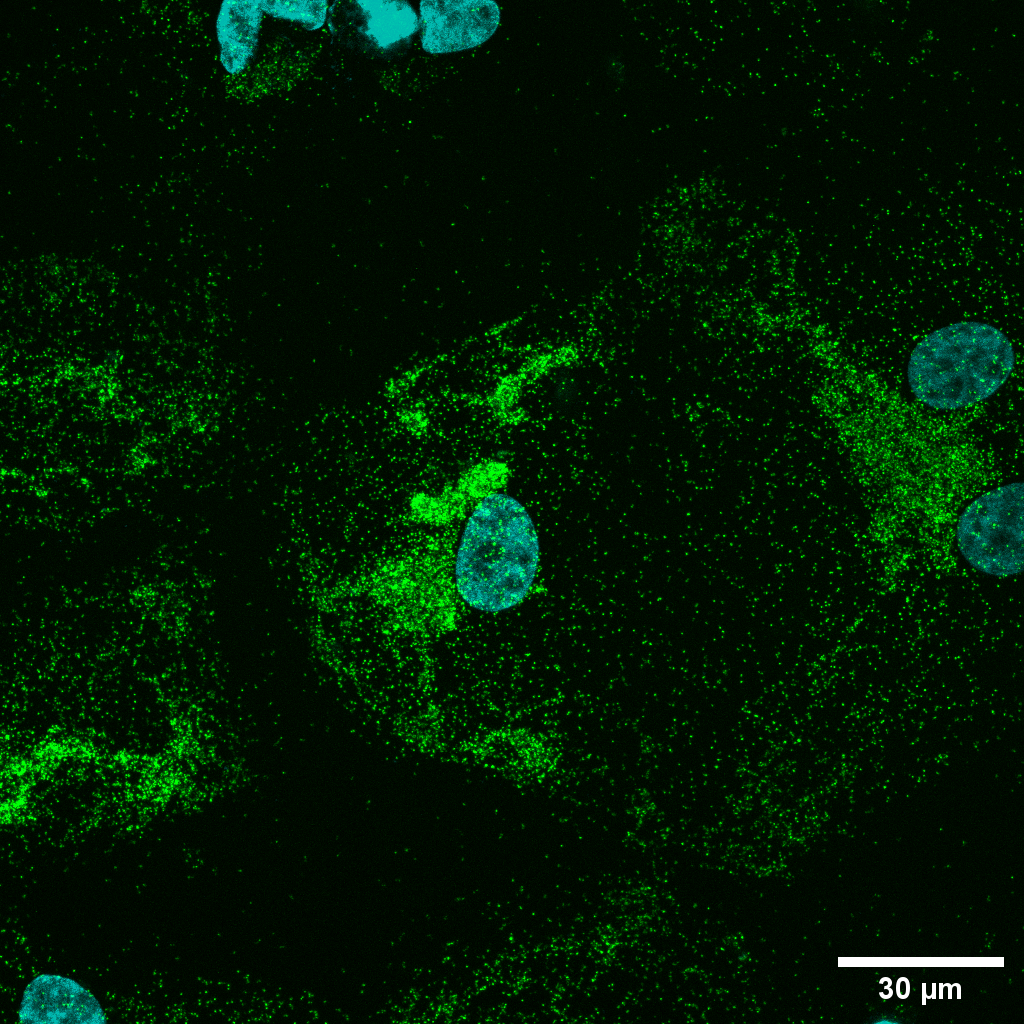

Supplement: Supplementary file 3 — Source data Fig. 1 [file 44321_2024_132_MOESM3_ESM.zip › Figure 1/1G/Max intensity_Mel null hiPSC-CMs_AAV6-Myc-Mel_Myc+α-MTP PLA.tif]

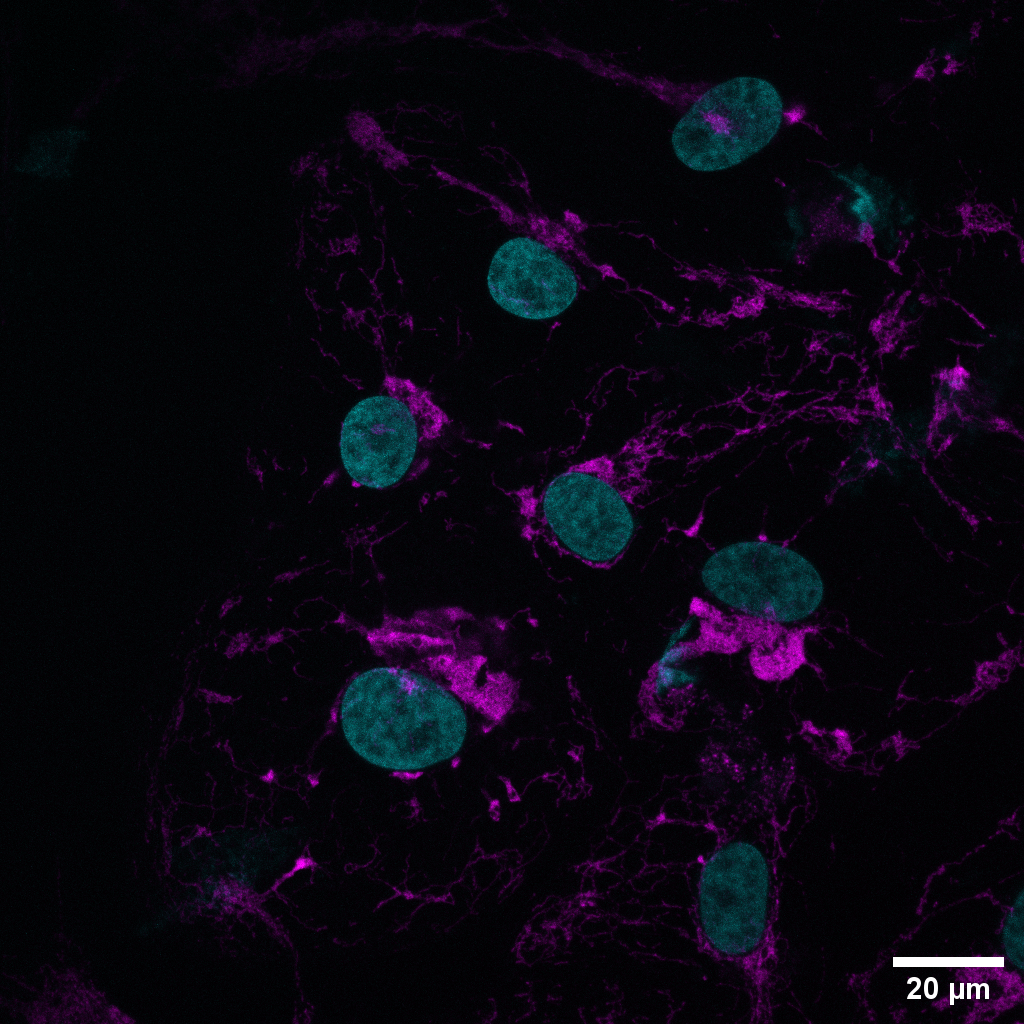

Supplement: Supplementary file 3 — Source data Fig. 1 [file 44321_2024_132_MOESM3_ESM.zip › Figure 1/1G/Max intensity_Mel null hiPSC-CMs_AAV6-Myc-Mel_α-MTP IF.tif]

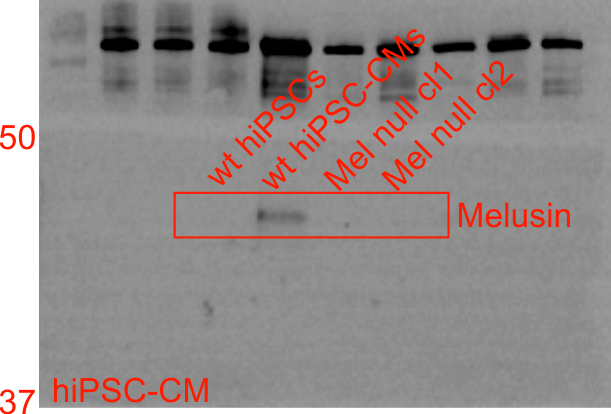

Supplement: Supplementary file 3 — Source data Fig. 1 [file 44321_2024_132_MOESM3_ESM.zip › Figure 1/1I/western melusin Mel null hiPSC-CMs.tif]

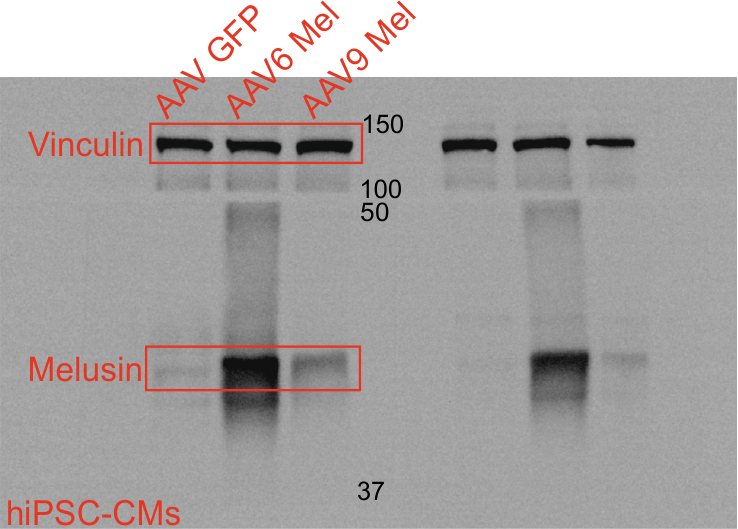

Supplement: Supplementary file 3 — Source data Fig. 1 [file 44321_2024_132_MOESM3_ESM.zip › Figure 1/1I/western vinculin + melusin AAV hiPSC-CMs.tif]

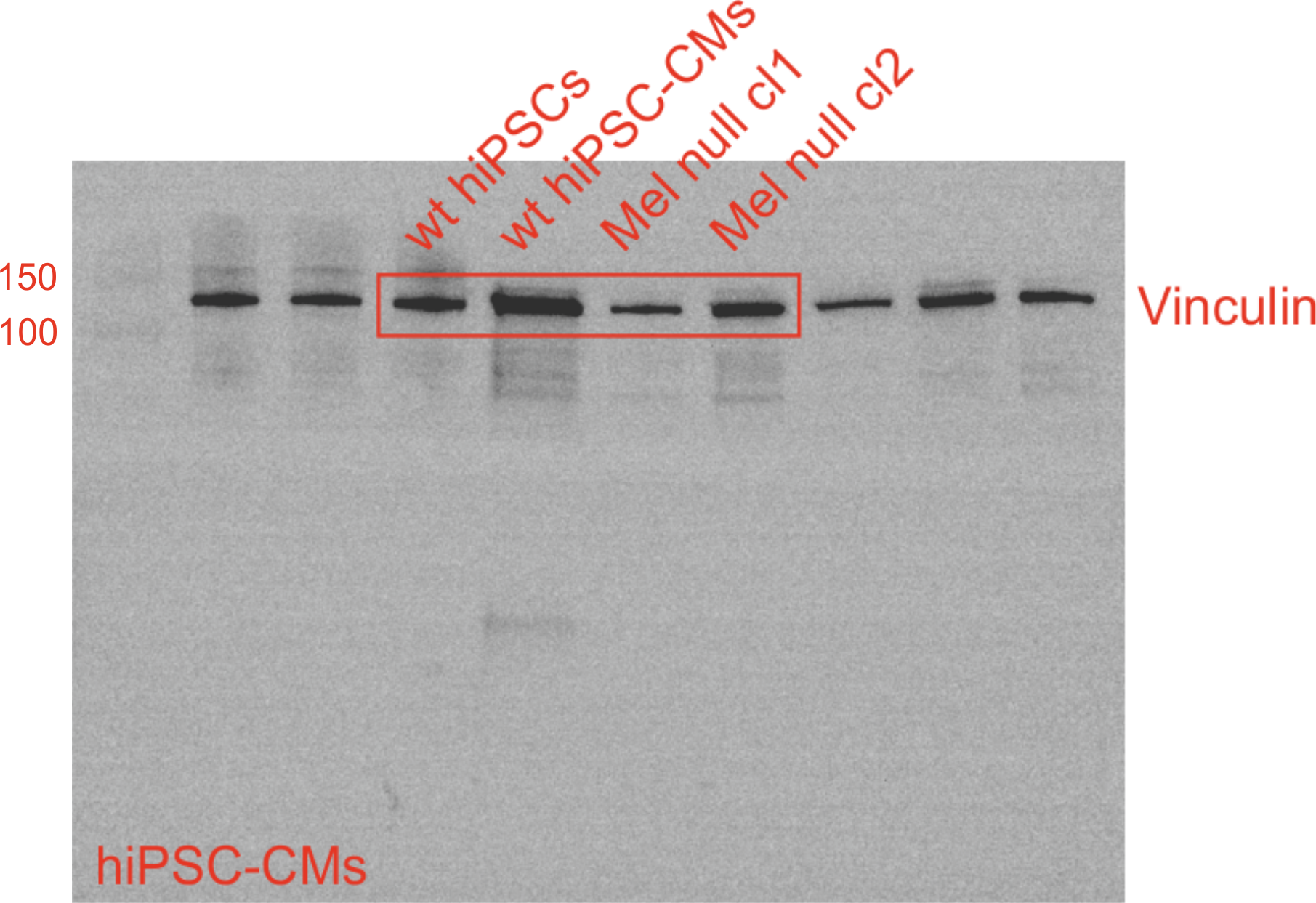

Supplement: Supplementary file 3 — Source data Fig. 1 [file 44321_2024_132_MOESM3_ESM.zip › Figure 1/1I/western vinculin Mel null hiPSC-CMs.tif]

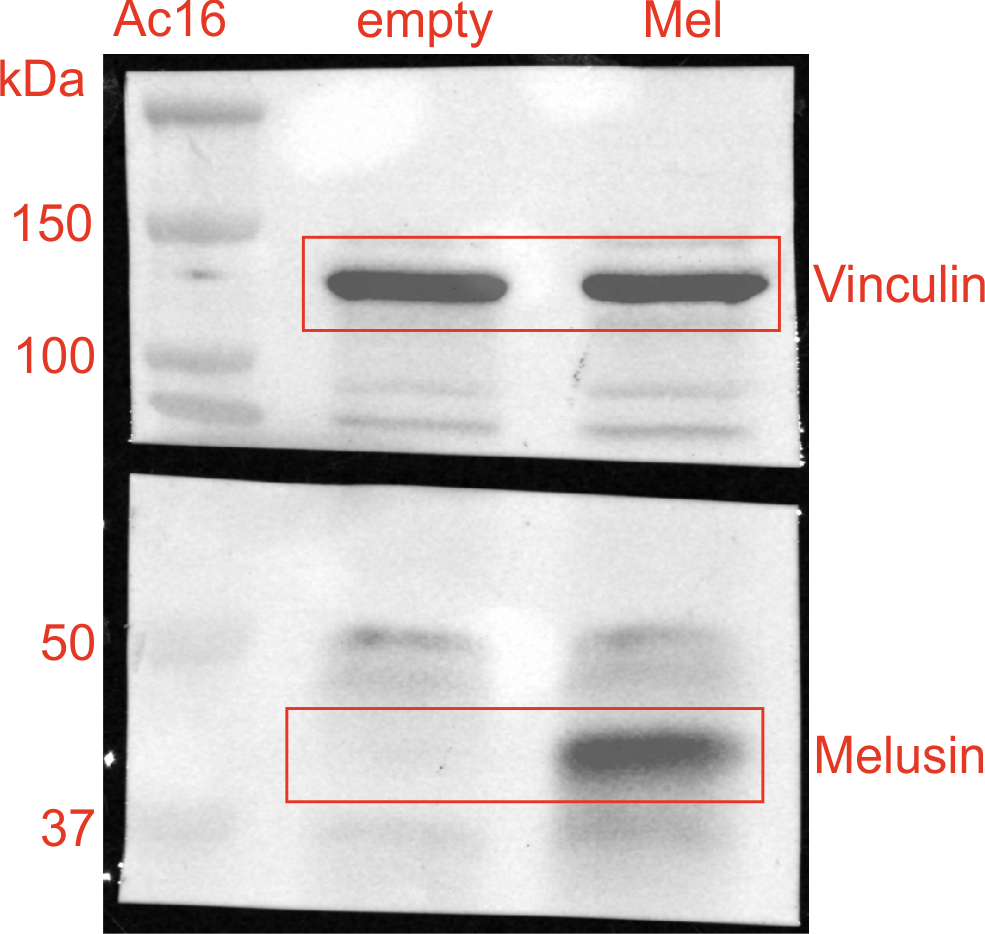

Supplement: Supplementary file 3 — Source data Fig. 1 [file 44321_2024_132_MOESM3_ESM.zip › Figure 1/1L/western vinculin + melusin AC16.tif]
