# Supplementary material for: An intrinsic mechanism of metabolic tuning promotes cardiac resilience to stress
Source: EMBO Mol Med. 2024 Sep 13;16(10):2450–84. doi: 10.1038/s44321-024-00132-z (PMC11473679; doi:10.1038/s44321-024-00132-z)
Supplement: Supplementary file 6 — Source data Fig. 4 [file 44321_2024_132_MOESM6_ESM.zip › Figure 4/4D/AC16 DOXO n1/JC1_AC16_DOXO_n1_BV.pdf]

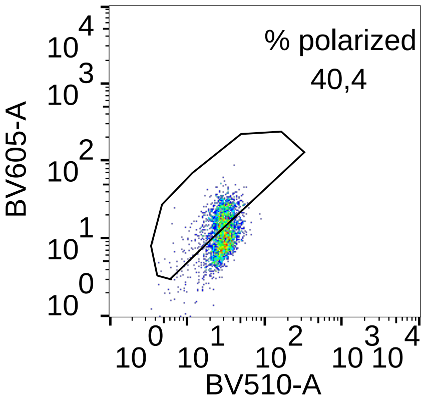

JC1_AC16_DOXO_001_AC16_EMPTY_DOXO_002_005.fcs
Single Cells
2951

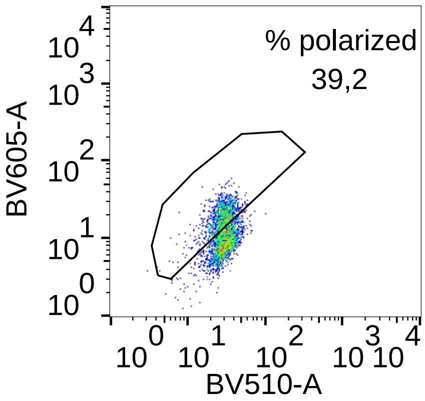

JC1_AC16_DOXO_001_AC16_EMPTY_DOXO_003_009.fcs
Single Cells
3223

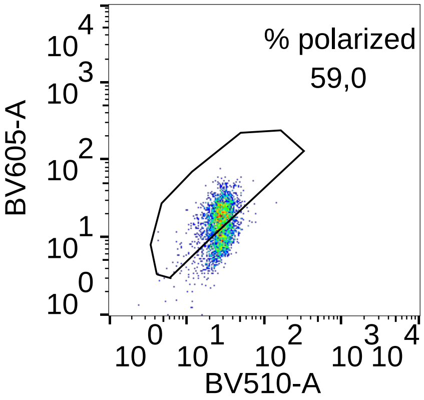

JC1_AC16_DOXO_001_AC16_MEL_DOXO_001_007.fcs
Single Cells
3242

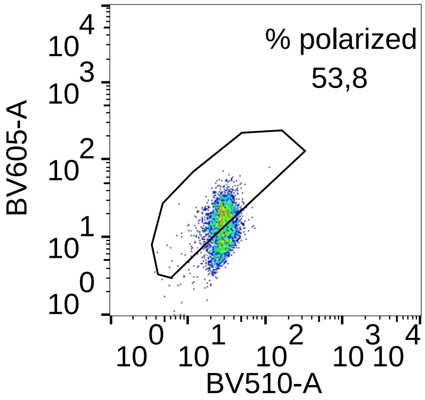

JC1_AC16_DOXO_001_AC16_MEL_DOXO_002_008.fcs
Single Cells
3719
