# Supplementary material for: An intrinsic mechanism of metabolic tuning promotes cardiac resilience to stress
Source: EMBO Mol Med. 2024 Sep 13;16(10):2450–84. doi: 10.1038/s44321-024-00132-z (PMC11473679; doi:10.1038/s44321-024-00132-z)
Supplement: Supplementary file 6 — Source data Fig. 4 [file 44321_2024_132_MOESM6_ESM.zip › Figure 4/4D/AC16 DOXO n2/JC1_AC16_DOXO_n2_SSC_FSC.pdf]

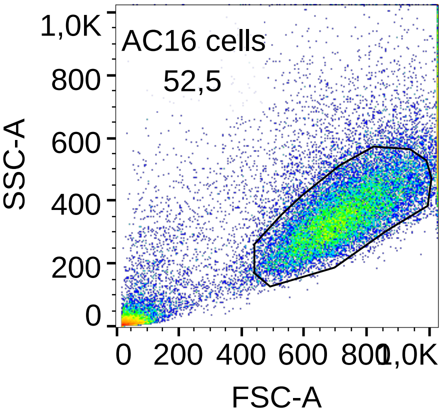

JC1_AC16_DOXO_003_AC16_EMPTY_DOXO_JC1_001_006.fc
Ungated
30000

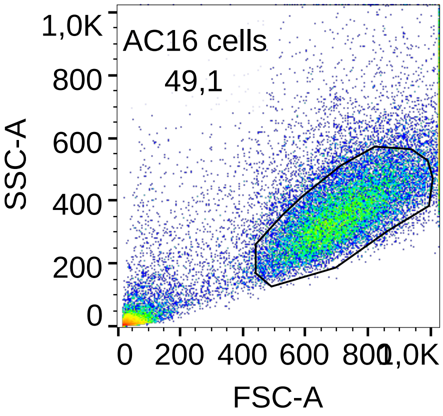

JC1_AC16_DOXO_003_AC16_MEL_DOXO_JC1_001_012.fc
Ungated
30000

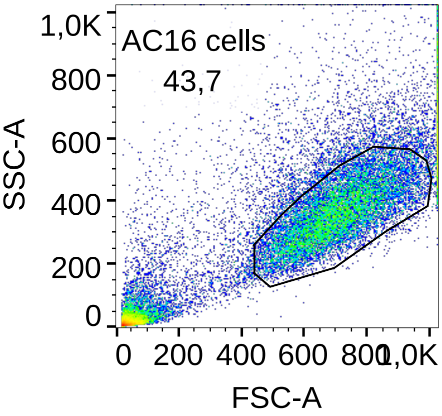

JC1_AC16_DOXO_003_AC16_EMPTY_DOXO_JC1_002_007.fc
Ungated
30000

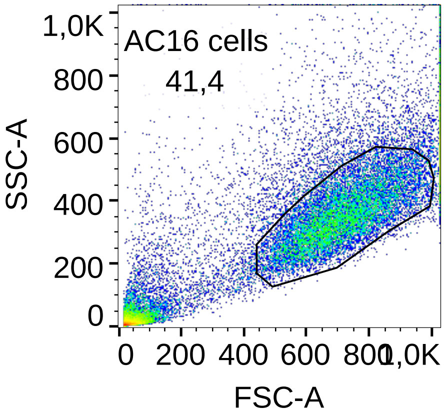

JC1_AC16_DOXO_003_AC16_MEL_DOXO_JC1_002_008.fc
Ungated
30000

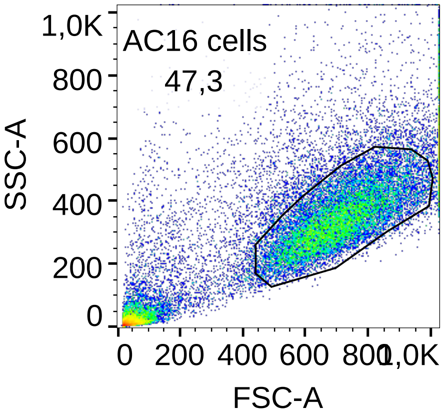

JC1_AC16_DOXO_003_AC16_EMPTY_DOXO_JC1_003_010.fc
Ungated
30000

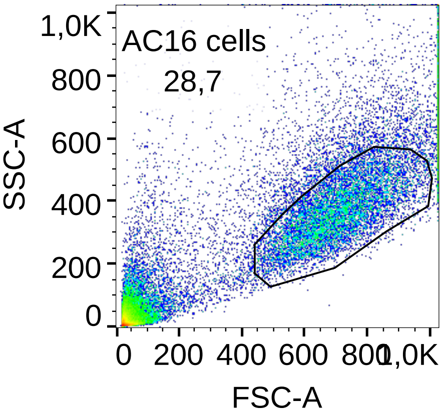

JC1_AC16_DOXO_003_AC16_MEL_DOXO_JC1_003_009.fc
Ungated
30000
