# Supplementary material for: An intrinsic mechanism of metabolic tuning promotes cardiac resilience to stress
Source: EMBO Mol Med. 2024 Sep 13;16(10):2450–84. doi: 10.1038/s44321-024-00132-z (PMC11473679; doi:10.1038/s44321-024-00132-z)
Supplement: Supplementary file 6 — Source data Fig. 4 [file 44321_2024_132_MOESM6_ESM.zip › Figure 4/4D/AC16 DOXO n3/JC1_AC16_DOXO_n3_SSC_FSC.pdf]

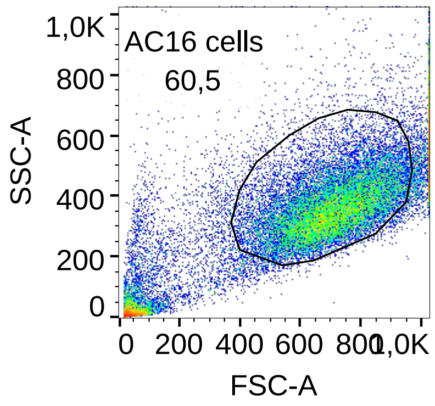

JC1_AC16_DOXO_006_AC16_empty_DOXO_001_006.fcs
Ungated
30000

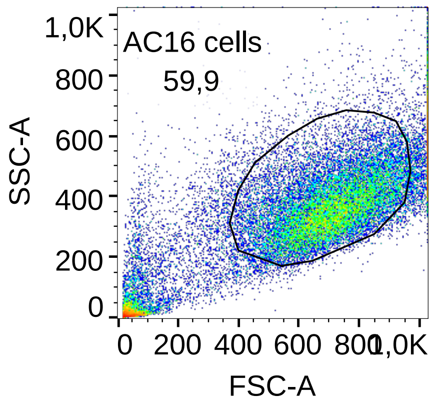

JC1_AC16_DOXO_006_AC16_MEL_DOXO_001_004.fcs
Ungated
30000

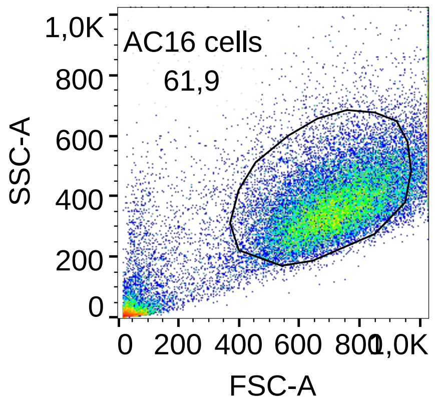

JC1_AC16_DOXO_006_AC16_empty_DOXO_002_007.fcs
Ungated
30000

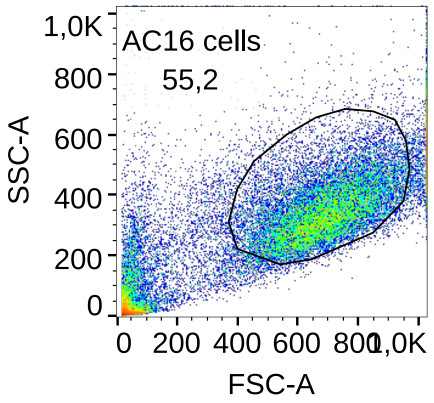

JC1_AC16_DOXO_006_AC16_MEL_DOXO_002_005.fcs
Ungated
30000
