# Supplementary material for: An intrinsic mechanism of metabolic tuning promotes cardiac resilience to stress
Source: EMBO Mol Med. 2024 Sep 13;16(10):2450–84. doi: 10.1038/s44321-024-00132-z (PMC11473679; doi:10.1038/s44321-024-00132-z)
Supplement: Supplementary file 6 — Source data Fig. 4 [file 44321_2024_132_MOESM6_ESM.zip › Figure 4/4E/JC1_CARDIO_DOXO_01_BV.pdf]

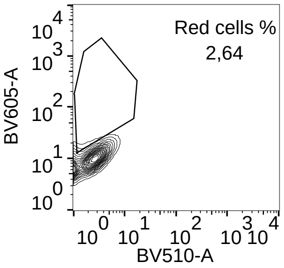

JC1_CARDIO_Test_03_KO_DOX_001_005.fcs
Single Cells
4732

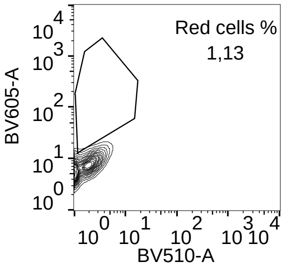

JC1_CARDIO_Test_03_KO_DOX_002_006.fcs
Single Cells
5320

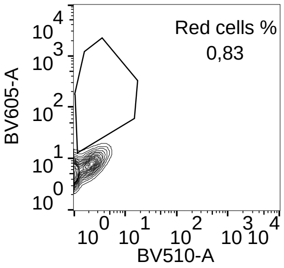

JC1_CARDIO_Test_03_KO_DOX_003_007.fcs
Single Cells
4822

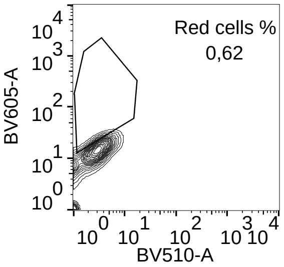

JC1_CARDIO_Test_03_UNSTAINED_DOXO_001.fcs
Single Cells
1602

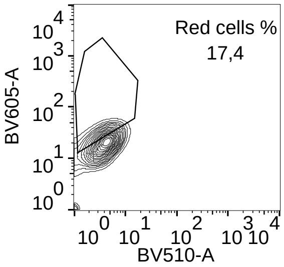

JC1_CARDIO_Test_03_WT_DOX_001_002.fcs
Single Cells
1454

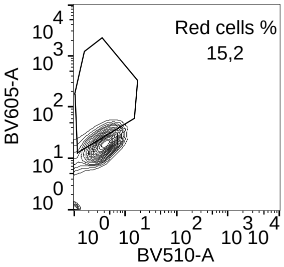

JC1_CARDIO_Test_03_WT_DOX_002_003.fcs
Single Cells
1993

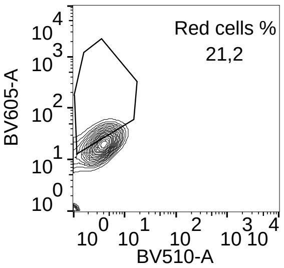

JC1_CARDIO_Test_03_WT_DOX_003_004.fcs
Single Cells
2014
