# Supplementary material for: An intrinsic mechanism of metabolic tuning promotes cardiac resilience to stress
Source: EMBO Mol Med. 2024 Sep 13;16(10):2450–84. doi: 10.1038/s44321-024-00132-z (PMC11473679; doi:10.1038/s44321-024-00132-z)
Supplement: Supplementary file 9 — Expanded View Figures [file 44321_2024_132_MOESM9_ESM.pdf]

Expanded View Figures

Figure EV1. Melusin binds the mitochondrial trifunctional protein.

(**A**) Coomassie Blue staining of Melusin immunoprecipitation (IP) from cardiac total extracts of Mel over mice and Mel null mice as controls. The indicated bands were cut out and identified by mass spectrometry. (**B**) Melusin immunoprecipitation from cardiac total extracts of wild-type mice and Mel null mice, as controls. The immunoprecipitated Melusin and the co-immunoprecipitated α-MTP and β-MTP were detected by immunostaining. Vinculin was stained to verify the complete removal of the total extract. Representative of $n = 3$ independent experiments. (**C**) Mitochondrial (Mito) and cytosolic (Cyto) fractions are differentially isolated from wild-type hearts. Melusin presence was evaluated by immunostaining. Vinculin and actin were stained as cytosolic markers, α-MTP and Vdac1 as mitochondrial markers. Representative of $n = 4$ independent experiments. (**D**) Mitochondria isolated from wild-type hearts treated with Proteinase K (PK), osmotic shock (OS), and Triton X-100 (TX100), or a combination of them, in order to digest proteins of different mitochondrial compartments. Melusin presence was evaluated by immunostaining. Pdh and α-MTP were stained as markers of the matrix compartment, Vdac1 as markers of the outer mitochondrial membrane. Representative of $n = 3$ independent experiments. (**E**) Fractions of cytosol (Cyto), subsarcolemmal (SS), and intermyofibrillar (IMF) mitochondria differentially isolated from wild-type hearts. Melusin presence was evaluated by immunostaining. α-MTP and Vdac1 were stained as mitochondrial markers. Representative of $n = 3$ independent experiments. (**F**) Complexes of different molecular weights were obtained as gel filtration fractions from the cardiac total extract of wild-type, Mel null and Mel over mice. Fractions were immunostained for α-MTP, β-MTP, and Melusin. Red arrows indicate the corresponding molecular weights. Representative of $n = 3$ independent experiments. IP immunoprecipitation, PK proteinase K, OS osmotic shock, Vinc vinculin, Vdac1 voltage-dependent anion-selective channel 1, Pdh pyruvate dehydrogenase, SS subsarcolemmal mitochondria, IMF intermyofibrillar mitochondria.

 © The Author(s)

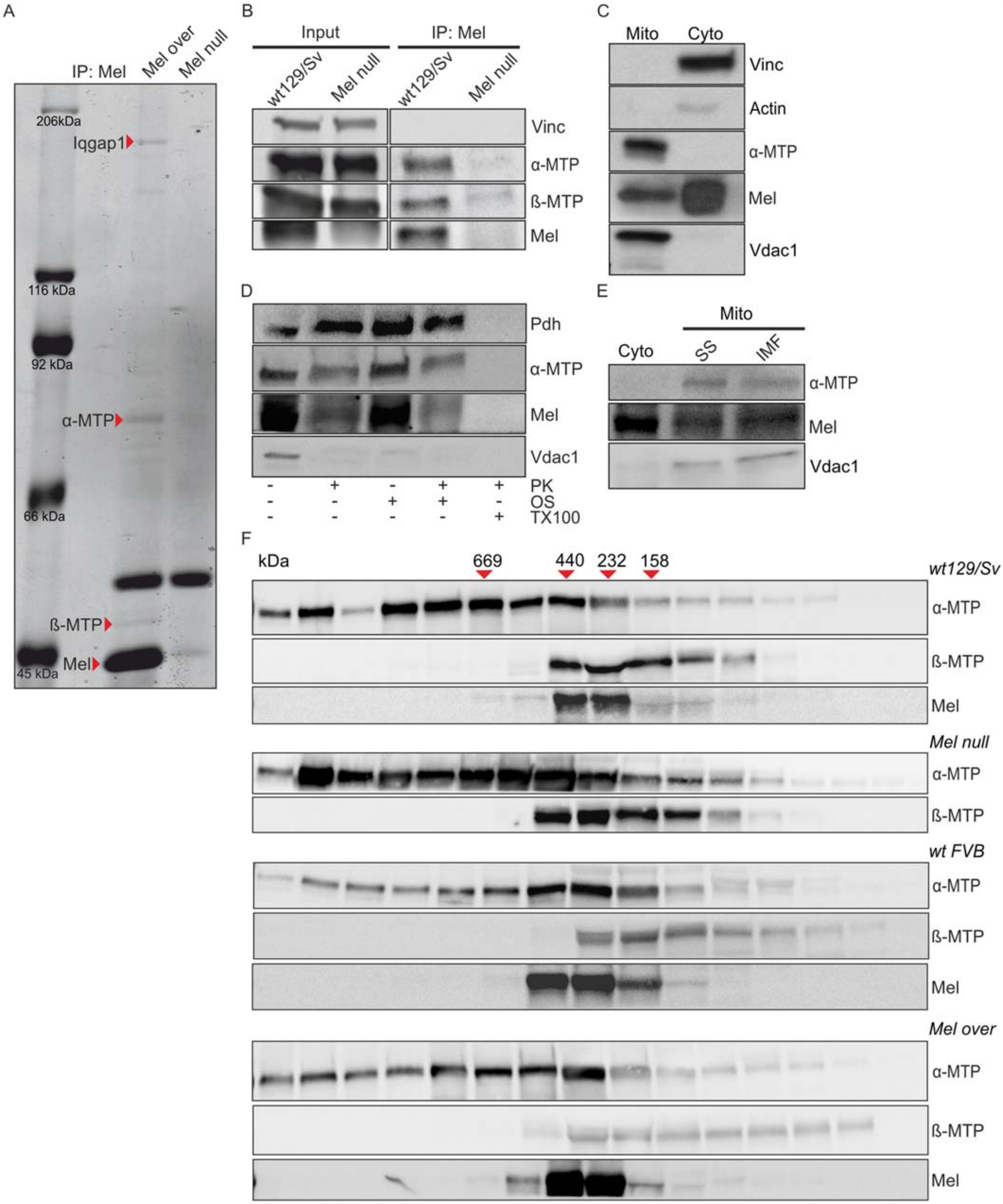
A
IP: Mel
Mel over
Mel null
206kDa
Iqgap1
116 kDa
92 kDa
α-MTP
66 kDa
ß-MTP
Mel
45 kDa
B
Input
IP: Mel
wt129/Sv
Mel null
wt129/Sv
Mel null
Vinc
α-MTP
ß-MTP
Mel
C
Mito
Cyto
Vinc
Actin
α-MTP
Mel
Vdac1
D
Pdh
α-MTP
Mel
Vdac1
PK
OS
TX100
E
Mito
Cyto
SS
IMF
α-MTP
Mel
Vdac1
F
kDa
669
440
232
158
wt129/Sv
α-MTP
ß-MTP
Mel
Mel null
α-MTP
ß-MTP
wt FVB
α-MTP
ß-MTP
Mel
Mel over
α-MTP
ß-MTP
Mel

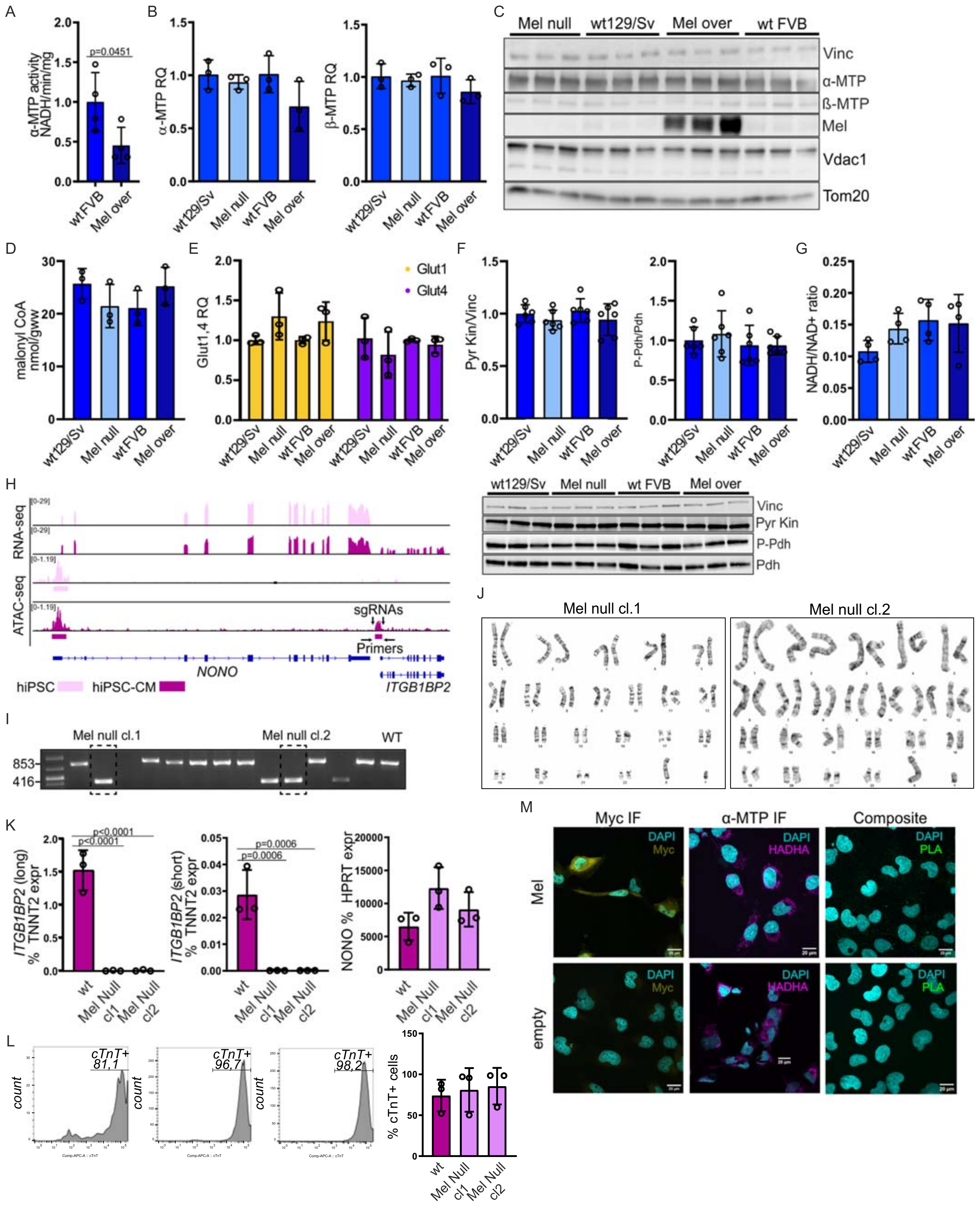
A
α-MTP activity NADH/min/mg
p=0.0451
wt FVB
Mel over
B
α-MTP RQ
β-MTP RQ
wt129/Sv
Mel null
wt FVB
Mel over
C
Mel null
wt129/Sv
Mel over
wt FVB
Vinc
α-MTP
ß-MTP
Mel
Vdac1
Tom20
D
malonyl CoA nmol/gww
E
Glut1,4 RQ
Glut1
Glut4
F
Pyr Kin/Vinc
P-Pdh/Pdh
G
NADH/NAD+ ratio
wt129/Sv
Mel null
wt FVB
Mel over
Vinc
Pyr Kin
P-Pdh
Pdh
H
RNA-seq
ATAC-seq
sgRNAs
Primers
NONO
ITGB1BP2
hiPSC
hiPSC-CM
I
Mel null cl.1
Mel null cl.2
WT
853
416
J
Mel null cl.1
Mel null cl.2
K
ITGB1BP2 (long) % TNNT2 expr
p<0.0001
p<0.0001
ITGB1BP2 (short) % TNNT2 expr
p=0.0006
p=0.0006
NONO % HPRT expr
wt
Mel Null cl1
Mel Null cl2
L
count
cTnT+ 81,1
cTnT+ 96,7
cTnT+ 98,2
% cTnT+ cells
M
Myc IF
α-MTP IF
Composite
Mel
empty
DAPI
Myc
HADHA
PLA

 © The Author(s)

Figure EV2. Characterization of MTP in mice with different expressions of Melusin and of Melusin-null hiPSCs.

(**A**) Modulation of α-MTP activity in wild-type and Mel over mice detected as NADH reduction in time in cardiac homogenates supplemented with acetoacetyl-CoA. ($n = 3$ per group; mean ± SD; p value by unpaired t-test; Data normalized on wt129/Sv media = 1). (**B**) Expression of α-MTP and β-MTP mRNA evaluated by real-time qPCR in hearts from Mel null, Mel over, and wild-type mice. ($n = 3$ per group; mean ± SD; p value by unpaired t-test). (**C**) Protein level of α-MTP, β-MTP, Vdac1, and Tom20 in cardiac extracts from Mel null, Mel over, and wild-type mice. Melusin was stained as a marker of the different genotypes. Vinculin was stained as a loading control. ($n = 3$ per group). (**D**) Malonyl-CoA level detected by ELISA assay in Mel null, Mel over, and wild-type hearts. ($n = 3$ per group; mean ± SD; p value by unpaired t-test). (**E**) Expression of Glut1 and Glut4 mRNA evaluated by real-time qPCR in hearts from Mel null, Mel over, and wild-type mice. ($n = 3$ per group; mean ± SD; p value by unpaired t-test). (**F**) Protein level of pyruvate kinase and phosphorylation ratio of Pdh in cardiac extracts from Mel null, Mel over, and wild-type mice. ($n = 6$ per group; mean ± SD; p value by unpaired t-test). (**G**) NADH/NAD+ ratio estimated by HPLC analysis in cardiac extracts from Mel null, Mel over, and wild-type mice. ($n = 4$ per group; mean ± SD; p value by unpaired t-test). (**H**) RNA-seq and ATAC-seq data of WT hESCs and hESC-CMs. *NONO* expression is constant (RNA-seq data) and the promoter region is accessible in both cell types (ATAC-seq data). *ITGB1BP2* promoter is accessible for transcription only in hiPSC-CMs. The sgRNA guides for Melusin knocking out were designed to bind the promoter region up- and down-stream the ATAC-seq peak. Primers for genotyping were designed against outside of the predicted cut sites. (**I**) Genotyping of Mel null hiPSC clones. The 853 bp band is the product of WT locus amplification, and the lower band (416 bp) is the result of promoter deletion. WT hiPSCs were used as control. All genome-edited clones show either the WT or promoter deletion band since Melusin is X-linked and the experiment involved male hiPSCs. (**J**) Karyotypes of Mel null cl.1 and cl.2 hiPSCs. Metaphase chromosomes were stained by G-banding. The karyotypes of Mel null hiPSCs are normal without clonal abnormalities and belong to a male individual. (**K**) RT-qPCR of WT, Mel null cl.1 and cl.2 hiPSC-CMs. *ITGB1BP2* mRNA was normalized on the muscle-specific marker *TNNT2*, so as to better account for variations in hiPSC-CM purity in samples analyzed prior to lactate selection. Two RefSeq isoforms for the *ITGB1BP2* gene were probed: the full-length canonical Melusin isoform (NM_012278.4) and a shorter isoform proved to be expressed at ~2% of Melusin (NM_001303277.3). The short isoform is predicted to share the same promoter as Melusin. Both isoforms were undetectable in both Mel null clones, validating the knockout strategy. To rule out a negative effect of the *ITGB1BP2* promoter deletion on the housekeeping *NONO* gene, located very close upstream of *ITGB1BP2*, *NONO* mRNA was analyzed and normalized on the housekeeping gene *HPRT*. ($n = 3$ total RNA isolation per group from hiPSC-CMs independently differentiated; mean ± SD; p value by one-way ANOVA with Bonferroni correction). (**L**), cTnT + cells by flow cytometry of WT, Mel null cl.1 and cl.2 hiPSC-CMs post lactate selection (histograms are gated based on isotype control stains), and relative graph. ($n = 3$ per group; each experiment is performed by using hiPSC-CMs independently differentiated; mean ± SD; p value by one-way ANOVA with Bonferroni correction). (**M**) Representative Immunofluorescence (IF) and PLA signal of Myc-Melusin (Myc-tag antibody) and α-MTP (HADHA antibody) in AC16 cells empty (empty) or expressing Myc-Melusin (Mel). DAPI was used to stain nuclei. Images were captured by confocal microscopy. Scale bare = 20 μm. Representative of $n = 3$ independent experiments. Vinc vinculin, Vdac1 voltage-dependent anion-selective channel 1, Tom20 translocase of outer membrane 20, Pyr Kin pyruvate kinase, P-Pdh phosphorylated pyruvate dehydrogenase, Pdh pyruvate dehydrogenase, sgRNAs single guide RNAs, *ITGB1BP2* Integrin β1 binding protein 2, bp base pair, *TNNT2* troponin type2, cTNT cardiac troponin T, *HPRT* hypoxanthine phosphoribosyltransferase 1, PLA proximity ligation assay.

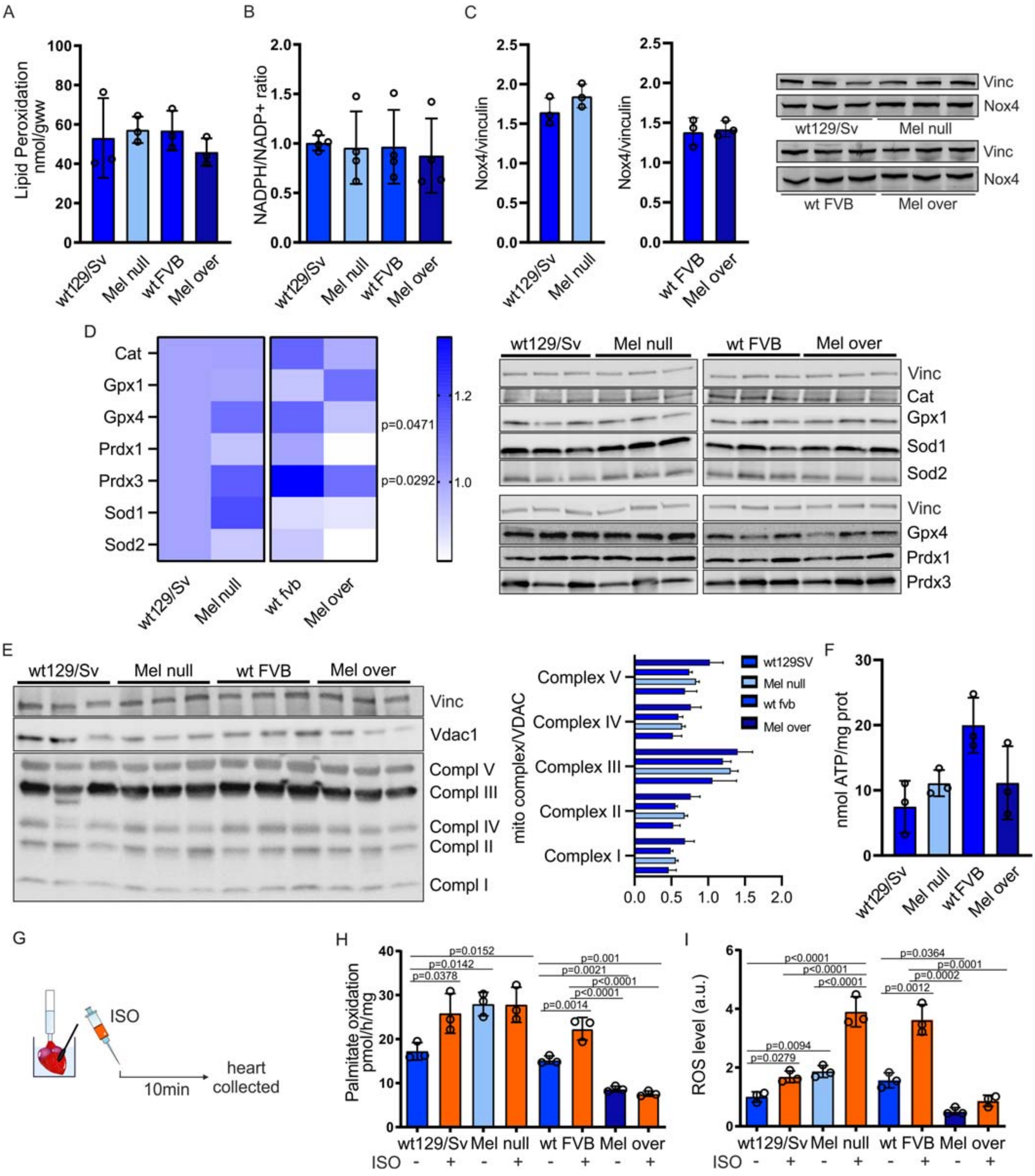
A
Lipid Peroxidation nmol/gww
wt129/Sv
Mel null
wt FVB
Mel over
B
NADPH/NADP+ ratio
C
Nox4/vinculin
Vinc
Nox4
D
Cat
Gpx1
Gpx4
Prdx1
Prdx3
Sod1
Sod2
p=0.0471
p=0.0292
wt fvb
Sod2
E
Vdac1
Compl V
Compl III
Compl IV
Compl II
Compl I
mito complex/VDAC
Complex V
Complex IV
Complex III
Complex II
Complex I
wt129SV
F
nmol ATP/mg prot
G
ISO
10min
heart collected
H
Palmitate oxidation pmol/h/mg
p=0.0152
p=0.0142
p=0.0378
p=0.001
p=0.0021
p<0.0001
p<0.0001
p=0.0014
I
ROS level (a.u.)
p<0.0001
p<0.0001
p<0.0001
p=0.0094
p=0.0279
p=0.0364
p=0.0001
p=0.0002
p=0.0012

 © The Author(s)

Figure EV3. Determination of parameters impacting mitochondrial function and ROS generation and evaluation of high-fat diet effect.

(**A**) Modulation of lipid peroxides detected as colorimetric reaction with malonaldehyde and 4-hydroxyalkenal in cardiac extracts of wild-type, Mel null, and Mel over mice. ($n = 3$ per group. mean ± SD; p value by unpaired t-test). (**B**) NADPH/NADP+ ratio estimated by HLPC analysis in cardiac extracts from Mel null, Mel over, and wild-type mice. ($n = 4$ per group; mean ± SD; p value by unpaired t-test). (**C**) Protein level of Nox4 in cardiac extracts from Mel null, Mel over, and wild-type mice in basal conditions (graphs of relative quantification (left) and immunostaining (right)). Vinculin was stained as a loading control. ($n = 3$ per group; mean ± SD; p value by unpaired t-test). (**D**) Heat map (left) and representative immunostaining (right) of the antioxidant enzymes catalase (Cat), glutathione peroxidase 1 (Gpx1), glutathione peroxidase 4 (Gpx4), peroxiredoxin 1 (Prdx 1), peroxiredoxin 3 (Prdx 3), superoxide dismutase 1 (Sod 1), superoxide dismutase 2 (Sod 2) in cardiac extracts from Mel null, Mel over, and wild-type mice. Vinculin was stained as a loading control. ($n = 6$ per group; mean ± SD; p value by unpaired t-test). (**E**) Left: Immunostaining for the five mitochondrial complexes in cardiac extracts from Mel null, Mel over, and wild-type mice. Vdac1 was stained as a marker of mitochondrial quantity. Vinculin was stained as a loading control. Right: relative quantifications. ($n = 3$ per group. mean ± SD; p value by unpaired t-test). (**F**) Level of ATP in total cardiac extracts of wild-type, Mel null, and Mel over mice, detected by luciferin-luciferase assay. ($n = 3$ per group; mean ± SD; p value by unpaired t-test). (**G**) Isoproterenol treatment protocol: hearts from Mel null, Mel over, and wild-type mice were excised and perfused ex-vivo with 1 µM isoproterenol (ISO +) or saline (ISO −), as control. (**H**) Modulation of palmitate oxidation, evaluated as generation of radioactive metabolites of $[1-^{14}C]$-palmitate, in cardiac isolated mitochondria from Mel null, Mel over, and wild-type mice treated as described in (**F**). ($n = 3$ per group; mean ± SD; p value by two-way ANOVA with Tukey correction (performed separately for the two mice strains)). (**I**) ROS content, measured as DCFDA-AM fluorescence, in cardiac isolated mitochondria from Mel null, Mel over, and wild-type mice treated as described in (**A**). Data normalized on wt129/Sv (wt129/Sv media = 1). ($n = 3$ per group; mean ± SD; p value by two-way ANOVA with Tukey correction (performed separately for the two mice strains)). Cat catalase, Gpx1 glutathione peroxidase 1, Gpx4 glutathione peroxidase 4, Prdx 1 peroxiredoxin 1, Prdx 3 peroxiredoxin 3, Sod 1 superoxide dismutase 1, Sod 2 Superoxide dismutase 2, Vinc vinculin, Vdac1 voltage-dependent anion-selective channel 1, ISO isoproterenol.

© The Author(s)

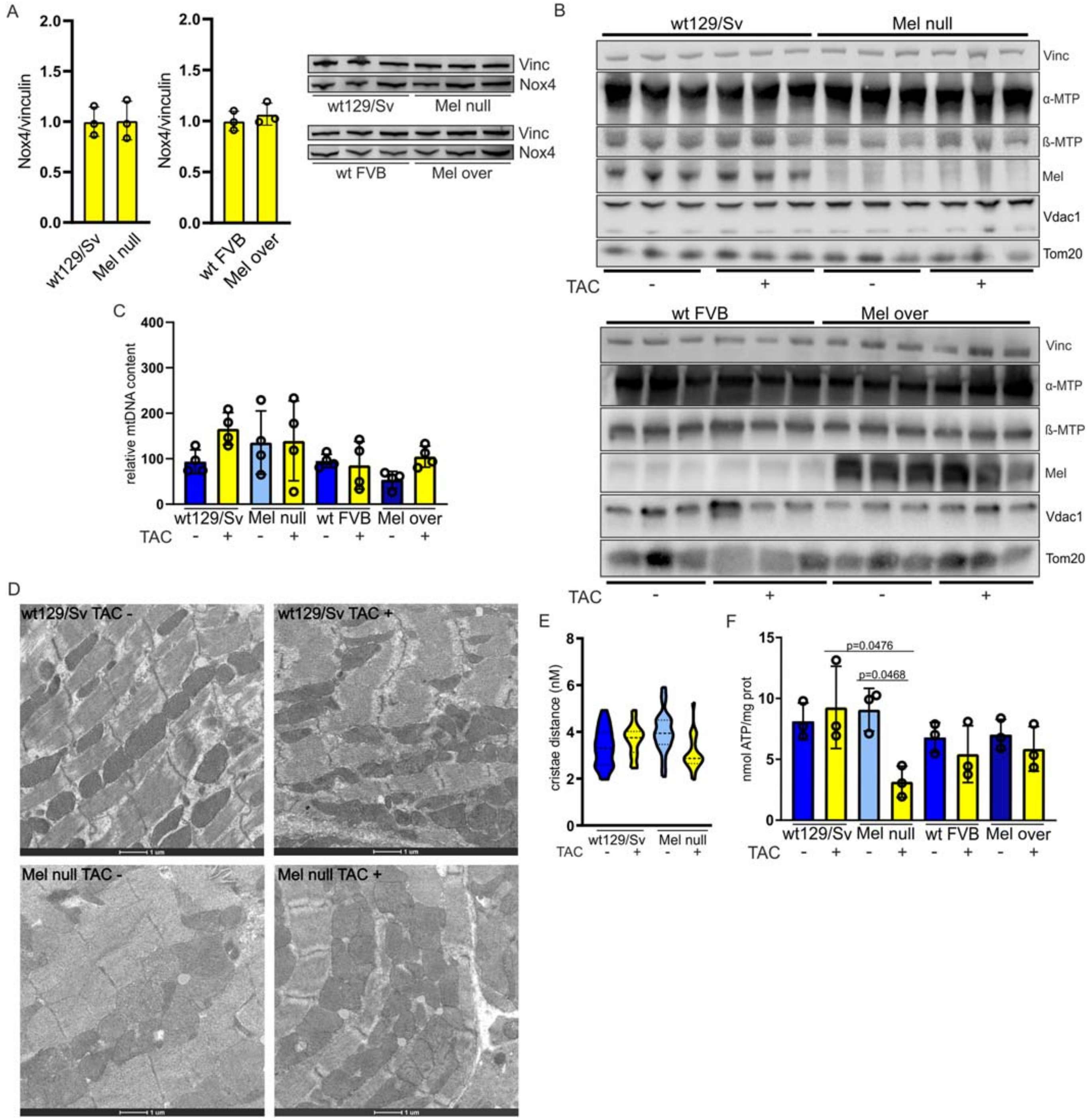
A
Nox4/vinculin
wt129/Sv
Mel null
wt FVB
Mel over
Vinc
Nox4
B
wt129/Sv
Mel null
Vinc
α-MTP
ß-MTP
Mel
Vdac1
Tom20
TAC
wt FVB
Mel over
C
relative mtDNA content
D
wt129/Sv TAC -
wt129/Sv TAC +
Mel null TAC -
Mel null TAC +
E
cristae distance (nM)
F
nmol ATP/mg prot
p=0.0476
p=0.0468

Figure EV4. Evaluation of protein expression and structural morphology of mitochondria from mice subjected to pressure overload.

(**A**) Protein level of Nox4 in cardiac extracts from Mel null, Mel over, and wild-type mice subjected to TAC surgery for 4 days (graphs of relative quantification (left) and immunostaining (right)). Vinculin was stained as a loading control. ($n = 3$ per group; mean ± SD; p value by unpaired t-test). (**B**) Protein level of α-MTP, β-MTP, Vdac1, and Tom20 in cardiac extracts from Mel null, Mel over, and wild-type mice subjected to TAC (TAC +) or sham (TAC −) surgery for 4 days, as described in 3D. Melusin was stained as a marker of the different genotypes. Vinculin was stained as a loading control. ($n = 3$ per group). (**C**) Relative mtDNA content compared to genomic DNA evaluated by quantitative real-time PCR analysis in hearts of wild-type, Mel null and Mel over mice subjected to TAC (TAC +) or sham (TAC −) surgery for 4 days, as described in 3D. ($n = 4$ per group; mean ± SD; p value by two-way ANOVA with Tukey correction (performed separately for the two mice strains)). (**D**) Representative TEM images of cardiac sections of wild-type and Mel null hearts subjected to TAC (TAC +) or sham (TAC −) surgery for 4 days, as described in 3D. Scale bare = 1 µm. (**E**) Relative quantification of cristae distance in nm. Data were means ± SD. ($n = 25$ cristae from mitochondria from three different animals per group. mean ± SD; p value by two-way ANOVA with Bonferroni correction). (**F**) Level of ATP, detected by luciferin-luciferase assay, in total cardiac extracts of wild-type, Mel null and Mel over mice subjected to TAC (TAC +) or sham (TAC −) surgery for 4 days, as described in 3D. ($n = 3$ per group; mean ± SD; p value by two-way ANOVA with Tukey correction (performed separately for the two mice strains)). Vinc vinculin; Vdac1 voltage-dependent anion-selective channel 1, Tom20 translocase of outer membrane 20, TAC transverse aortic constriction, TEM transmission electron microscopy.

© The Author(s)

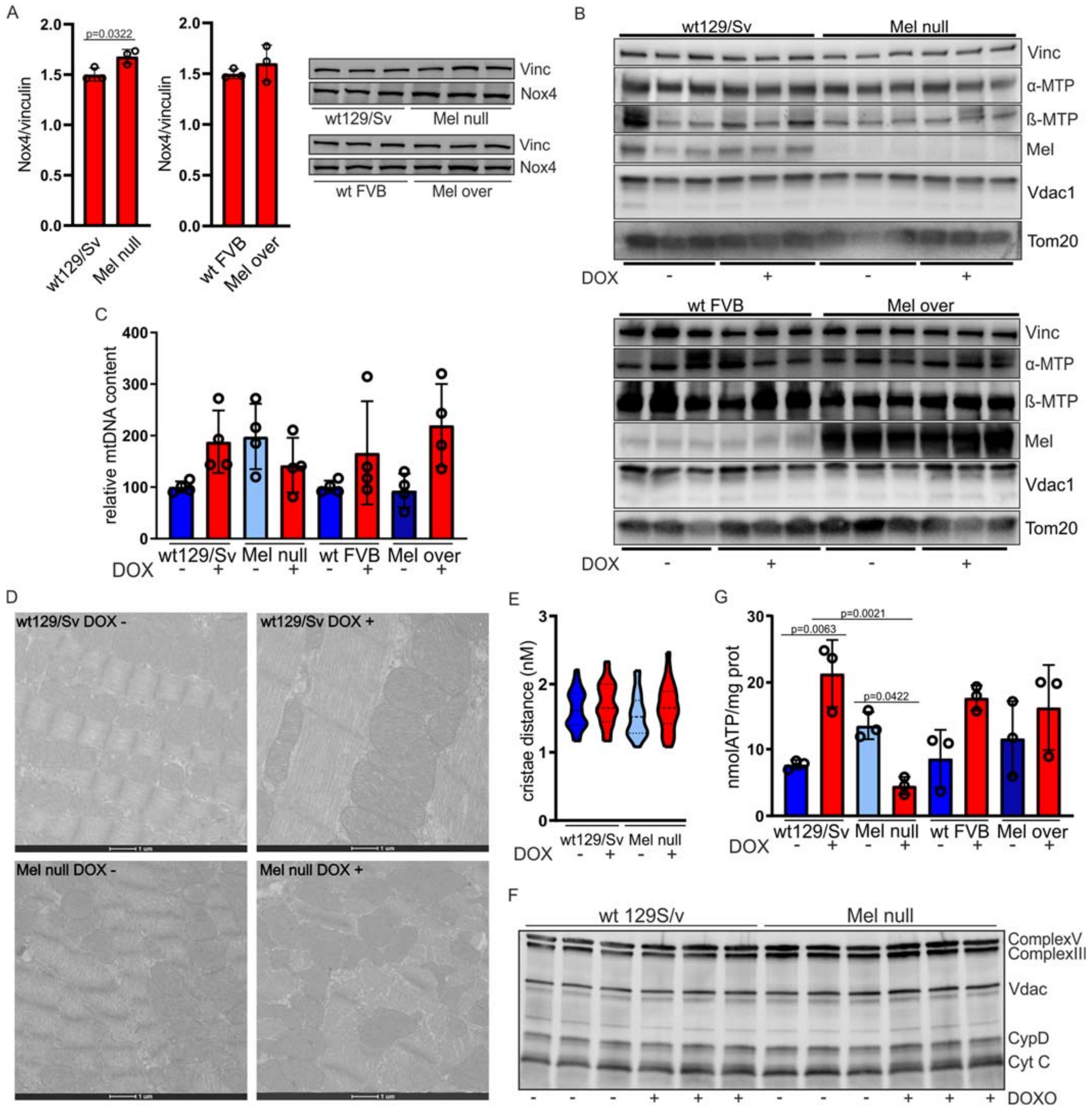
A
Nox4/vinculin
p=0.0322
wt129/Sv
Mel null
wt FVB
Mel over
Vinc
Nox4
B
wt129/Sv
Mel null
Vinc
α-MTP
ß-MTP
Mel
Vdac1
Tom20
DOX
wt FVB
Mel over
C
relative mtDNA content
DOX
D
wt129/Sv DOX -
wt129/Sv DOX +
Mel null DOX -
Mel null DOX +
E
cristae distance (nM)
G
nmolATP/mg prot
p=0.0063
p=0.0021
p=0.0422
F
wt 129S/v
Mel null
ComplexV
ComplexIII
Vdac
CypD
Cyt C
DOXO

Figure EV5. Evaluation of protein expression and structural morphology of mitochondria from mice subjected to doxorubicin cardiotoxicity.

(**A**) Protein level of Nox4 in cardiac extracts from Mel null, Mel over, and wild-type mice treated with 4 mg/kg doxorubicin for 6 h (graphs of relative quantification (left) and immunostaining (right)). Vinculin was stained as a loading control. ($n = 3$ per group; mean ± SD; p value by unpaired t-test). (**B**) Protein level of α-MTP, β-MTP, Vdac1, and Tom20 in cardiac extracts from Mel null, Mel over, and wild-type mice treated with 4 mg/kg doxorubicin (DOX +) or saline (DOX −) for 6 h, as described in 3**F**. Melusin was stained as a marker of the different genotypes. Vinculin was stained as a loading control. ($n = 3$ per group). (**C**) Relative mtDNA content compared to genomic DNA evaluated by quantitative real-time PCR analysis in hearts of wild-type, Mel null, and Mel over mice treated with 4 mg/kg doxorubicin (DOX +) or saline (DOX −) for 6 h, as described in 3**F**. ($n = 4$ per group; mean ± SD; p value by two-way ANOVA with Tukey correction (performed separately for the two mice strains)). (**D**) Representative TEM images of cardiac sections of wild-type and Mel null mice treated with 4 mg/kg doxorubicin (DOX +) or saline (DOX −) for 6 h, as described in 3**F**. Scale bar = 1 µm. (**E**) Relative quantification of cristae distance in nm. Data were means ± SD. ($n = 25$ cristae from mitochondria from three different animals per group. mean ± SD; p value by two-way ANOVA with Bonferroni correction). (**F**) Integrity of isolated mitochondria from wild-type 129S/v and Mel null hearts, untreated or treated with doxorubicin, estimated as immunostaining of specific markers of different mitochondrial compartments (Complex Va, Complex III for the inner membrane, Vdac for the outer membrane, cyclophilin D for the matrix, cytochrome C for the intermembrane space. ($n = 3$ per group). (**G**) Level of ATP, detected by luciferin-luciferase assay, in total cardiac extracts of wild-type, Mel null and Mel over mice treated with 4 mg/kg doxorubicin (DOX +) or saline (DOX −) for 6 h, as described in 3F. ($n = 3$ per group; mean ± SD; p value by two-way ANOVA with Tukey correction (performed separately for the two mice strains)). Vinc vinculin, Vdac voltage-dependent anion-selective channel, Tom20 translocase of outer membrane 20, DOX doxorubicin, TEM transmission electron microscopy, CypD cyclophilin D, Cyt C cytochrome C.

© The Author(s)
